# Dihydroartemisinin targets the miR-497-5p/SOX5 axis to suppress tumor progression in non-small cell lung cancer

**DOI:** 10.3389/fphar.2025.1605531

**Published:** 2025-07-08

**Authors:** Qing-Hua Yin, Qiang Zhou, Jian-Bing Hu, Jie Weng, Er-Dong Shen, Fang Wen, Song-Lian Liu, Lei-Lan Yin, Ya-Jun Tong, Ling Long, Ke-Wei Tang, Si-Te Bai, Lu-Di Ou

**Affiliations:** Department of Oncology, Yueyang Central Hospital, Yueyang, Hunan, China

**Keywords:** dihydroartemisinin (DHA), non-small cell lung cancer (NSCLC), miR-497-5p, SOX5, anticancer activity

## Abstract

**Introduction:**

Non-small cell lung cancer (NSCLC) remains a lethal malignancy with limited therapeutic options. Although dihydroartemisinin (DHA) exhibits anticancer properties, its mechanisms in NSCLC are incompletely understood. This study investigated the role of the miR-497-5p/SOX5 axis in mediating DHA’s effects on NSCLC.

**Methods:**

*In vitro* experiments utilized A549 and H1299 cells treated with DHA (50 μM). Proliferation, migration, invasion, and apoptosis were assessed. miR-497-5p and SOX5 expression was modulated via genetic silencing. *In vivo*, A549 xenograft tumor growth in mice was evaluated under DHA treatment (25/50 mg/kg).

**Results:**

DHA significantly suppressed proliferation, migration, and invasion while inducing apoptosis *in vitro*. Mechanistically, DHA upregulated miR-497-5p and downregulated SOX5—overexpressed in clinical NSCLC. Silencing miR-497-5p attenuated DHA’s effects and increased SOX5, whereas SOX5 knockdown reversed miR-497-5p inhibition. *In vivo*, DHA dose-dependently inhibited tumor growth with miR-497-5p elevation and SOX5 suppression, effects abrogated by miR-497-5p inhibition but rescued by SOX5 knockdown.

**Discussion:**

DHA exerts antitumor activity by activating the miR-497-5p/SOX5 axis, revealing a novel mechanism. Bridging efficacious *in vitro* concentrations with clinically achievable dosing remains essential for therapeutic translation.

## 1 Introduction

Non-small cell lung cancer (NSCLC) represents one of the most prevalent and lethal malignancies worldwide, accounting for approximately 85% of all lung cancer cases (Siegel et al., 2023). Despite significant advancements in early diagnosis and targeted therapies, the 5-year survival rate for NSCLC patients remains disappointingly low, particularly for those with advanced-stage disease (Herbst et al., 2018). Current treatment modalities, including surgical resection, chemotherapy, radiotherapy, and immunotherapy, remain the cornerstone of NSCLC management. However, these approaches are often limited by their efficacy and associated toxicities (Hirsch et al., 2017). For instance, while chemotherapeutic agents can inhibit tumor growth, their nonspecific cytotoxicity to normal tissues poses significant challenges for clinical application. Therefore, identifying novel therapeutic targets and agents is critical to improving outcomes for NSCLC patients.

MicroRNAs (miRNAs) are a class of small non-coding RNAs, approximately 22 nucleotides in length, that play pivotal roles in regulating gene expression and modulating various biological processes (Bartel, 2004). Accumulating evidence suggests that miRNAs are deeply involved in the initiation, progression, and metastasis of NSCLC (He et al., 2020). Certain miRNAs can function as either tumor suppressors or oncogenes, influencing critical cellular processes such as proliferation, apoptosis, migration, and invasion (Shenouda and Alahari, 2009; Singh et al., 2025; Lobera et al., 2023). Among these, miR-497-5p has recently garnered attention for its tumor-suppressive properties (Huang et al., 2019; Tang et al., 2022). Studies have demonstrated that miR-497-5p is downregulated in NSCLC and exerts its anti-tumor effects by targeting multiple signaling pathways (Li G. et al., 2019). However, the precise regulatory mechanisms of miR-497-5p in NSCLC and its potential therapeutic implications remain incompletely understood.

Dihydroartemisinin (DHA), a derivative of artemisinin, is well-known for its potent antimalarial and anticancer properties (Dai et al., 2021; Olatunde et al., 2023). Recent studies have revealed that DHA exhibits remarkable anti-tumor effects in various cancer models, including the inhibition of cell proliferation, induction of apoptosis, and suppression of metastasis (Lu and Efferth, 2021). In NSCLC, DHA has been shown to modulate multiple signaling pathways, such as PI3K/AKT, MAPK, and Wnt/β-catenin, to exert its anticancer activity (Rani et al., 2025). Nevertheless, whether DHA exerts its effects through the regulation of miRNA expression in NSCLC remains poorly explored. Furthermore, the potential relationship between DHA and miR-497-5p, as well as the underlying mechanisms, warrants further investigation.

This study aims to elucidate whether dihydroartemisinin inhibits NSCLC cell proliferation, apoptosis, migration, and metastasis by targeting the miR-497-5p/SOX5 axis. By integrating molecular biology experiments and animal models, we provide the first evidence that DHA exerts its anti-tumor effects by upregulating miR-497-5p expression and suppressing the SOX5 signaling pathway. Our findings not only offer novel insights into the therapeutic potential of DHA in NSCLC but also pave the way for the development of miRNA-based targeted therapies.

## 2 Methods

### 2.1 Patient tissue specimens

Paraffin-embedded tissue specimens investigated in this study were retrospectively collected from the archival repository of Yueyang Central Hospital. Samples were obtained from five non-small cell lung cancer (NSCLC) patients who underwent resection surgery between 2024.1 and 2025.1. Written informed consent for tissue specimen usage was obtained from all participants. The study protocol received approval from the Institutional Review Board (Ethics Committee) of Yueyang Central Hospital.

### 2.2 Immunohistochemistry (IHC)

Immunohistochemical analysis was performed to investigate SOX5 expression in lung adenocarcinoma (LAC). Tissue sections were deparaffinized in xylene (3 × 5 min) and rehydrated through graded alcohols: absolute ethanol (3 × 3 min), 95% ethanol (3 × 3 min), and 70% ethanol (3 min). Sections were subsequently immersed in distilled H_2_O and rinsed with phosphate-buffered saline (PBS; 3 × 5 min).

Endogenous peroxidase activity was quenched by incubating sections in 3% H_2_O_2_/PBS solution for 10 min, followed by H_2_O rinsing. Antigen retrieval was performed using target retrieval solution under high-temperature conditions in an autoclave (5 min at boiling). After PBS rinses (3 × 5 min), sections were blocked with an avidin/biotin blocking solution supplemented with 10% normal goat serum in PBS for 30 min.

Sections were incubated with appropriately diluted primary antibodies and biotin solution overnight at 4°C. Primary antibodies included: GAPDH (1:5000; ab181602, Abcam),SOX5 (1:1000; ab94396, Abcam),E-cadherin (1:1000; ab40772, Abcam),N-cadherin (1:1000; ab76011, Abcam),MMP-2 (1:1000; ab92536, Abcam),MMP-9 (1:1000; ab76003, Abcam),Bax (1:1000; ab182733, Abcam),Bcl-2 (1:1000; ab182858, Abcam).Following PBS washes (3 × 5 min), sections were incubated with biotinylated secondary antibody (1:200 dilution) for 30 min. After additional PBS rinses (3 × 5 min), detection was performed using Vectastain Elite ABC Reagent (Vector Labs, PK-6101) for 30 min. After final PBS washes (3 × 5 min), peroxidase activity was visualized using ImmPACT peroxidase substrate (Vector Labs, SK-4100) until optimal staining intensity developed. Sections were counterstained, dehydrated, cleared, and coverslipped. Stained sections were examined and digitally scanned using an Olympus microscope.

#### 2.2.1 IHC assessment

IHC results were evaluated independently by two blinded pathologists. Staining intensity in carcinoma cells was scored as: 0 (negative), 1 (weak), 2 (moderate), or 3 (strong). The percentage of stained tumor cells was scored as: 0 (0%), 1 (1%–20%), 2 (21%–40%), 3 (41%–60%), 4 (61%–80%), or 5 (81%–100%). A histologic score (H-score) was calculated as the product of intensity and percentage scores. Specimens were categorized as follows: H-score <2 indicated low expression; H-score ≥2 indicated high expression.

### 2.3 Cell culture

Human non-small cell lung cancer (NSCLC) cell lines A549 and H1299, as well as the normal human bronchial epithelial cell line BEAS-2B, were obtained from the Cell Bank of the Chinese Academy of Sciences. These cell lines were cultured in RPMI-1640 medium (HyClone, South Logan, UT) supplemented with 10% fetal bovine serum (FBS; Gibco, Carlsbad, CA), 100 U/mL penicillin, and 100 μg/mL streptomycin in a humidified incubator containing 5% CO_2_ at 37°C. Dihydroartemisinin (DHA) was purchased from MedChemExpress (Monmouth Junction, NJ, USA). DHA was dissolved in dimethyl sulfoxide (DMSO) to prepare solutions at concentrations of 5, 10, 25, 50, and 100 µM. The NSCLC cell lines were treated with DHA at these concentrations for 24, 48, and 72 h.

### 2.4 Cell transfection

The miR-497-5p inhibitor, mimic, and their respective negative controls (miR-NC inhibitor), as well as small interfering RNA (siRNA) targeting SOX5 (si-SOX5) and non-targeting siRNA (si-NC), were purchased from Genepharma (Shanghai, China). These oligonucleotides were transfected into A549 cells using Lipofectamine 2000 reagent (Invitrogen, Carlsbad, CA, USA) according to the manufacturer’s instructions. After 24 h of incubation, cells were collected for further experiments.

### 2.5 Cell viability assay

Cells treated with DHA were cultured for 24 h at 37°C in 5% CO_2_. Subsequently, 10 µL of Cell Counting Kit-8 (CCK-8) solution (Beyotime, Shanghai, China) was added to the cells. After a 4-h incubation, the absorbance of each well was measured at 450 nm using a DR-200Bs microplate reader (Wuxi Hiwell-Diatek Instruments Co., Ltd., China), with background absorbance corrected using a blank control. Data were analyzed to calculate cell viability or cytotoxicity, and corresponding proliferation or toxicity curves were plotted.

### 2.6 Flow cytometry

Cells were collected by trypsinization and centrifugation, washed with ice-cold phosphate-buffered saline (PBS), and resuspended in 1× binding buffer. The Annexin V-FITC/PI Apoptosis Detection Kit (BD Pharmingen, San Diego, CA, USA) was used to assess cell apoptosis according to the manufacturer’s instructions. Apoptotic cells were measured using a flow cytometer (BD Biosciences).

### 2.7 Wound healing assay

Cells were seeded at a density of 2.5 × 10^4^ cells per well in a 6-well plate. After the cells formed a continuous monolayer, they were starved for 12 h. A scratch was created using a 10 µL pipette tip, and floating cell debris was removed with PBS. Wound healing was observed and photographed under a microscope, and the scratch closure ratio was calculated to evaluate cell migration ability.

### 2.8 Transwell assay

Cell migration and invasion were assessed using Transwell chambers (Corning Inc.). For invasion assays, Transwell chambers were pre-coated with Matrigel (Corning Inc.). RPMI-1640 medium containing 10% FBS was added to the lower chamber, while 100 µL of serum-free medium-treated A549 cells were seeded into the upper chamber. The procedure was performed according to the manufacturer’s instructions. Cells that migrated to the lower surface of the upper chamber were fixed with paraformaldehyde (PFA; Sigma) and stained with crystal violet. The number of migrated cells was analyzed under a microscope.

### 2.9 Western blot analysis

Cells were washed three times with PBS, and total protein was extracted using RIPA buffer (Solarbio, Beijing, China). Protein concentration was determined using the BCA method. Proteins were separated by sodium dodecyl sulfate-polyacrylamide gel electrophoresis (SDS-PAGE) and transferred onto polyvinylidene fluoride (PVDF) membranes. After blocking with skimmed milk at 37°C for 2 h, membranes were incubated with primary antibodies overnight at 4°C. Secondary antibodies (1:2000) were incubated at room temperature for 30 min. GAPDH was used as an internal control. Membranes were treated with ECL substrate and exposed to film in a dark room. ImageJ software was used for densitometric analysis. Primary antibodies included: GAPDH antibody (1:5000; ab181602, Abcam), SOX5 antibody (1:1000; ab94396, Abcam), E-cadherin antibody (1:1000; ab40772, Abcam), N-cadherin antibody (1:1000; ab76011, Abcam), MMP-2 antibody (1:1000; ab92536, Abcam), MMP-9 antibody (1:1000; ab76003, Abcam), Bax antibody (1:1000; ab182733, Abcam), Bcl-2 antibody (1:1000; ab182858, Abcam).

### 2.10 Quantitative real-time PCR (qRT-PCR)

Total RNA was extracted using an RNA extraction kit (TransGen Biotech, Beijing, China). cDNA was synthesized using a cDNA synthesis kit, and qRT-PCR was performed according to the manufacturer’s instructions. Relative expression levels were calculated using the 2^−^ΔΔCt method. GAPDH and U6 were used as internal controls. Primer sequences are listed below:

U6: Forward, 5ʹ-CTC​GCT​TCG​GCA​GCA​CAT-3ʹ; Reverse, 5ʹ-AAC​GCT​TCA​CGA​ATT​TGC​GT-3ʹ.

miR-497-5p: Forward, 5ʹ-CAG​CAG​CAC​ACT​GTG​GTT​TGT-3ʹ; Reverse, 5ʹ-CTC​AAC​TGG​TGT​CGT​GGA​GTC-3ʹ.

GAPDH: Forward, 5ʹ-CAT​CAT​CCC​TGC​CTC​TAC​TGG-3ʹ; Reverse, 5ʹ-GTG​GGT​GTC​GCT​GTT​GAA​GTC-3ʹ.

SOX5: Forward, 5ʹ-CAG​ATG​GAG​AGG​TAG​CCA​TGG-3ʹ; Reverse, 5ʹ-CCA​TTG​TAT​TGT​GCT​GAG​AAG​TG-3ʹ.

E-Cad: Forward, 5ʹ-GAC​ACT​GGT​GCC​ATT​TCC​AC-3ʹ; Reverse, 5ʹ-AGT​TCG​AGG​TTC​TGG​TAT​GGG-3ʹ.

N-Cad: Forward, 5ʹ-GCA​ACG​ACG​GGT​TAG​TCA​CC-3ʹ; Reverse, 5ʹ-GAC​ACG​GTT​GCA​GTT​GAC​TGA​G-3ʹ.

MMP-2: Forward, 5ʹ-CCG​CAG​TGA​CGG​AAA​GAT​GT-3ʹ; Reverse, 5ʹ-CTT​GGT​GTA​GGT​GTA​AAT​GGG​TG-3ʹ.

MMP-9: Forward, 5ʹ-TCG​AAC​TTT​GAC​AGC​GAC​AAG-3ʹ; Reverse, 5ʹ-TCA​GTG​AAG​CGG​TAC​ATA​GGG​T-3ʹ.

Bax: Forward, 5ʹ-TCT​GAG​CAG​ATC​ATG​AAG​ACA​GG-3ʹ; Reverse, 5ʹ-ATC​CTC​TGC​AGC​TCC​ATG​TTA​C-3ʹ.

Bcl-2: Forward, 5ʹ-AGG​ATT​GTG​GCC​TTC​TTT​GAG-3ʹ; Reverse, 5ʹ-AGC​CAG​GAG​AAA​TCA​AAC​AGA​G-3ʹ.

### 2.11 Animal experiments

Male BALB/c mice (4–6 weeks old) were randomly assigned to eight groups (*n* = 3 per group). A549 cells (2 × 10^4^) were subcutaneously injected into the right flank of each mouse. Mice in the treatment groups received DHA (25 mg/kg or 50 mg/kg) via intragastric injection daily. Tumor volumes were measured every 7 days. After 2 weeks, mice were euthanized by cervical dislocation. The absence of spontaneous breathing and blink reflex within 2–3 min was considered indicative of death. Excised tumor xenografts were weighed. All animal studies were approved by the Ethics Committee of Yueyang Central Hospital and conducted in accordance with the ARRIVE guidelines and the Basel Declaration. Animals were humanely cared for in accordance with the guidelines of the National Institutes of Health (NIH).

### 2.12 Statistical analysis

All data were validated by three independent biological replicates and analyzed using GraphPad Prism 9.0 software (GraphPad Inc., La Jolla, CA, USA). Data are presented as mean ± standard deviation. One-way analysis of variance (ANOVA) was used for multiple group comparisons, with pairwise comparisons performed using the LSD-t test. Differences were considered statistically significant at *p* < 0.05.

## 3 Results

### 3.1 Dihydroartemisinin modulates viability, apoptosis, migration, and invasion in non-small cell lung cancer cells

**FIGURE 1:**
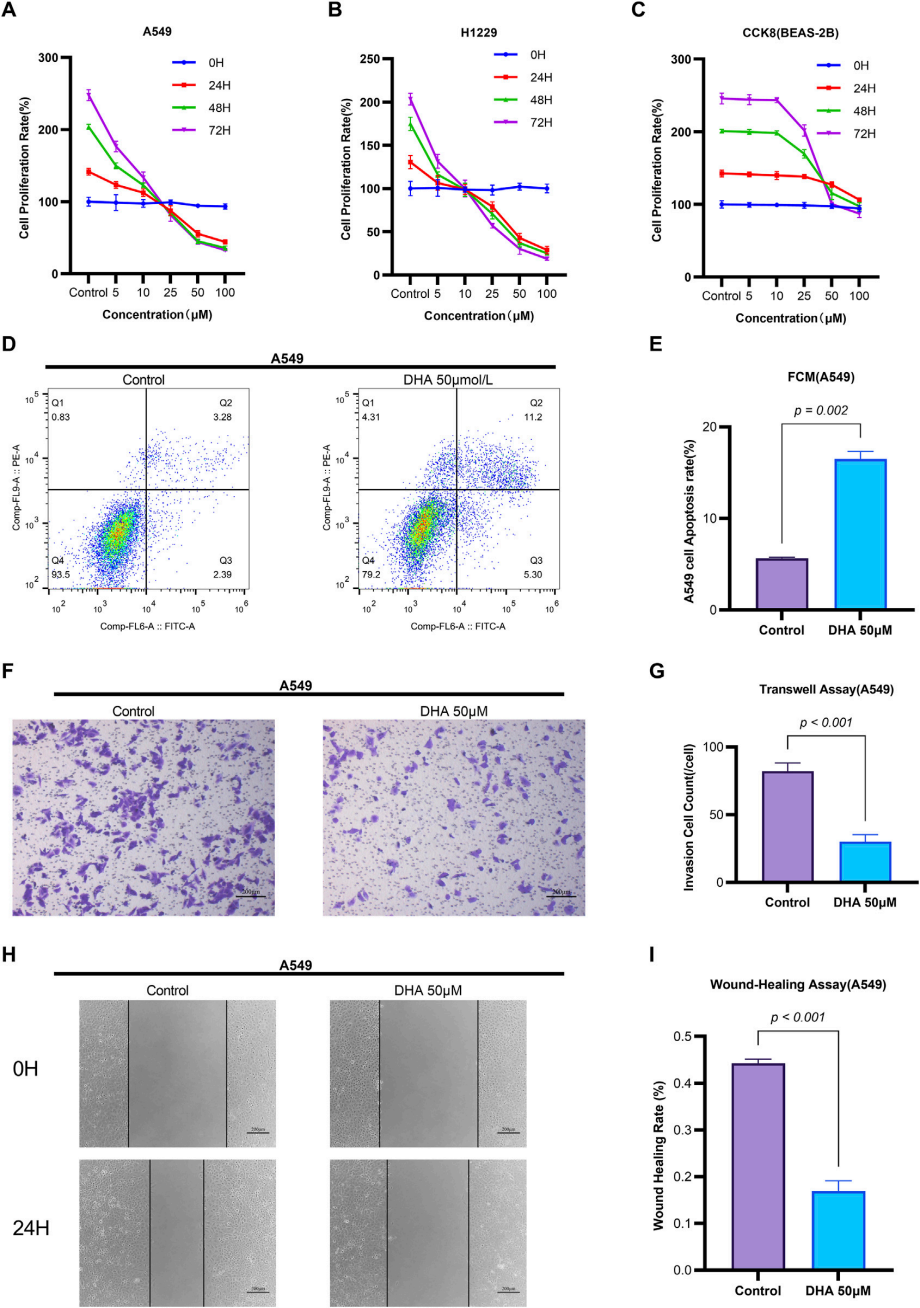
Dihydroartemisinin Modulates Viability, Apoptosis, Migration, and Invasion in Non-Small Cell Lung Cancer Cells. **(A–C)** Concentration- and time-dependent suppression of NSCLC cell viability by DHA CCK-8 assay demonstrating viability of A549 **(A)**, H1299 **(B)** and BEAS-2B **(C)** cells treated with DHA (0, 5, 10, 25, 50, 100 µM) for 24, 48, and 72 h **(D–E)** Induction of apoptosis by DHA treatment **(D)** Flow cytometry analysis of Annexin V/PI staining in A549 cells after 24 h exposure to 50 µM DHA. Quantification in **(E)** shows significant increase in total apoptosis (early + late apoptotic cells). **(F–G)** Inhibition of invasive capacity by DHA. **(F)** Representative images of Transwell invasion assay (Matrigel-coated) in A549 cells treated with 50 µM DHA for 24 h. **(G)** Quantitative analysis showing >60% reduction in invaded cells. **(H,I)** Suppression of migratory capacity by DHA. **(H)** Wound healing assay images at 0 and 24 h post-treatment with 50 µM DHA. **(I)** Quantification of wound closure rate demonstrating significant inhibition of migration. Each column represented the mean ± standard deviation (SD) (*n* = 3 fields per group).

To investigate the impact of dihydroartemisinin (DHA) on non-small cell lung cancer (NSCLC) progression, we assessed the proliferation rates of H1299 and A549 cells following treatment with varying concentrations of DHA (0–100 μM) for 24, 48, and 72 h using CCK-8 assays. DHA treatment induced a concentration- and time-dependent decrease in cellular proliferation (Figures 1A,B). In contrast, treatment with identical DHA concentrations and durations did not significantly inhibit the growth of BEAS-2B cells (normal human bronchial epithelial cells) (Figure 1C). These findings are consistent with established NSCLC models: reported IC_50_ values include 42.2 μM for A549 cells at 24 h (Wen et al., 2024) and 53.14 μM for LLC cells (Wang et al., 2024), confirming efficacy at concentrations >30 μM. Doses ≤30 μM demonstrate negligible cytotoxicity in these models (Jiang et al., 2016). Critically, DHA exhibited significant selectivity (Tong et al., 2016), as no toxicity was observed in normal bronchial epithelial cells (BEAS-2B) at therapeutic concentrations (Li et al., 2021). Based on this evidence, a concentration of 50 μM DHA was selected for subsequent experiments to ensure:(i) Robust anti-cancer activity (exceeding the IC_50_ threshold), (ii) Mechanistic relevance (minimal confounding effects from sub-therapeutic doses), and(iii) Safety profile (well below the cytotoxic threshold for normal cells).

**FIGURE 2:**
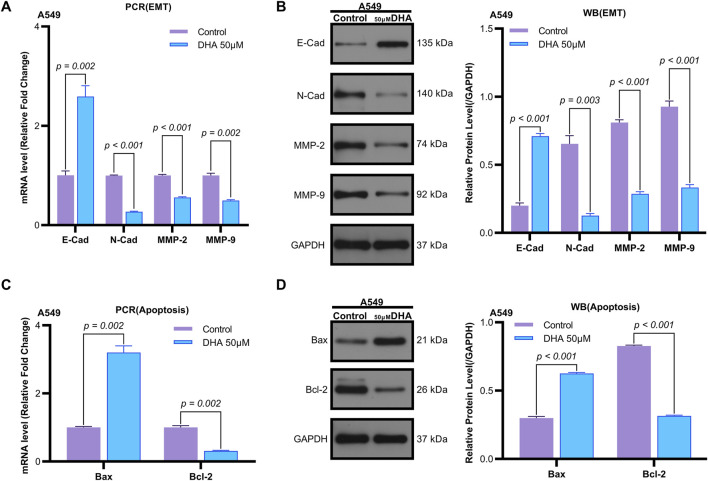
Dihydroartemisinin Modulates Expression of Epithelial-Mesenchymal Transition and Apoptosis Markers in A549 Cells. **(A)** mRNA expression levels of epithelial-mesenchymal transition (EMT) markers (E-cadherin, N-cadherin, matrix metalloproteinase-2 [MMP-2], matrix metalloproteinase-9 [MMP-9]) determined by quantitative real-time PCR (qRT-PCR) in A549 cells exposed to 50 µM dihydroartemisinin for 24 h. DHA significantly upregulated E-cadherin (*p* = 0.002) while downregulating N-cadherin (*p* < 0.001), MMP-2 (*p* < 0.001), and MMP-9 (*p* = 0.002) mRNA expression. **(B)** Western blot validation of EMT and apoptosis marker protein expression in A549 cells exposed to 50 µM dihydroartemisinin for 24 h. Protein-level changes corresponded with transcriptional alterations, showing elevated E-cadherin (*p* < 0.001) and reduced N-cadherin (*p* = 0.003), MMP-2 (*p* < 0.001), and MMP-9 (*p* < 0.001). **(C)** mRNA expression levels of apoptosis markers (Bax, Bcl-2) determined by qRT-PCR. DHA significantly upregulated Bcl-2 (*p* = 0.002) while downregulating Bax (*p* = 0.002) mRNA expression. **(D)** Protein expression levels of apoptosis markers (Bax, Bcl-2) determined by Western blot. Protein-level changes corresponded with transcriptional alterations, showing elevated Bax (*p* < 0.001) and reduced Bcl-2 (*p* < 0.001). GAPDH/β-actin served as loading controls for all experiments. Data represent mean ± SD of three independent experiments.

Flow cytometry analysis revealed that 50 μM DHA significantly induced apoptosis in A549 cells after 24 h of treatment (*p* = 0.002, Figures 1D,E). Quantitative reverse transcription PCR (qRT-PCR) demonstrated that DHA treatment upregulated the mRNA expression of the pro-apoptotic marker Bax (*p* = 0.002) and downregulated the anti-apoptotic marker Bcl-2 (*p* = 0.002) (Figure 2C). Western blot analysis further confirmed these results, showing a significant increase in Bax protein levels (*p* < 0.001) and a decrease in Bcl-2 protein levels (*p* < 0.001) (Figure 2D). Collectively, these findings indicate that DHA suppresses cell viability and promotes apoptosis in A549 cells.

Transwell assays were employed to evaluate the effects of DHA on cell migration and invasion. Treatment with 50 μM DHA for 24 h significantly reduced both migratory (Figures 1H,I, *p* < 0.001) and invasive (Figures 1F,G, *p* < 0.001) capacities of A549 cells compared to the control group. qRT-PCR analysis of epithelial-mesenchymal transition (EMT) markers revealed that DHA treatment increased E-Cadherin expression (*p* = 0.002) while decreasing N-Cadherin, MMP-2, and MMP-9 expression (N-Cad: *p* < 0.001; MMP2: *p* < 0.001; MMP9: *p* = 0.002) (Figure 2A). Western blot analysis corroborated these results, showing elevated E-Cadherin (*p* < 0.001) and reduced N-Cadherin (*p* = 0.003), MMP-2 (*p* < 0.001), and MMP-9 (*p* < 0.001) protein levels (Figure 2B). These data suggest that DHA inhibits migration and invasion in A549 cells.

### 3.2 Dihydroartemisinin upregulates miR-497-5p, and silencing miR-497-5p attenuates its anti-cancer effects in A549 cells

**FIGURE 3:**
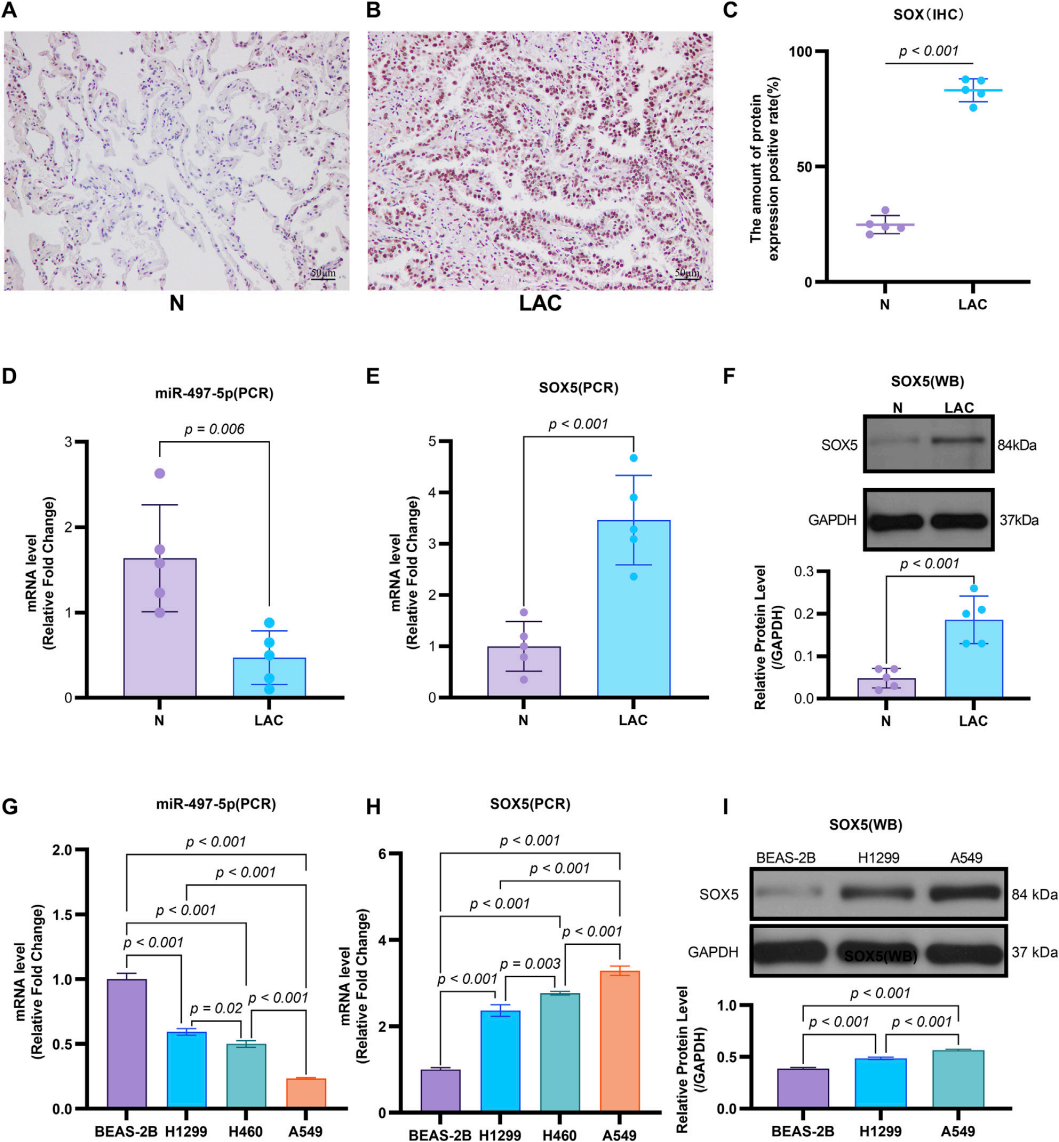
The Expression of miR-497-5p and SOX5 in LAC cells and tissues. **(A–C)** SOX5 is over-expressed in LAC tissues: Immunohistochemical analysis of SOX5 levels in five human lung adenocarcinoma patients (LAC) and their respective non-tumor counterparts (N), SOX5 was localized in the cytoplasm **(A,B)**. SOX5 expression is significantly higher in LAC than in non-tumor counterparts **(C)**. **(D)** Quantitative RT-PCR analysis of miR-497-5p mRNA level in the same patients, normalized versus U6. **(E)** Quantitative RT-PCR analysis of SOX5 mRNA level in the same patients, normalized versus U6. **(F)** Western blot analysis of SOX5 levels in four human lung adenocarcinoma patients (LAC) and their respective non-tumor counterparts (N). *p* < 0.001 (LAC vs. N). **(G)** Quantitative RT-PCR analysis of miR-497-5p level in several lung cancer cell lines (H1299, H460 and A549) and normal human bronchial epithelial cells (BEAS-2B). Data normalized to U6 snRNA. **(H)** Quantitative RT-PCR analysis of SOX5 level in several lung cancer cell lines (H1299, H460 and A549) and normal human bronchial epithelial cells (BEAS-2B). Data normalized to U6 snRNA. **(I)** Western blot analysis of SOX5 level several lung cancer cell lines (H1299, H460 and A549). GAPDH served as endogenous control.

**FIGURE 4:**
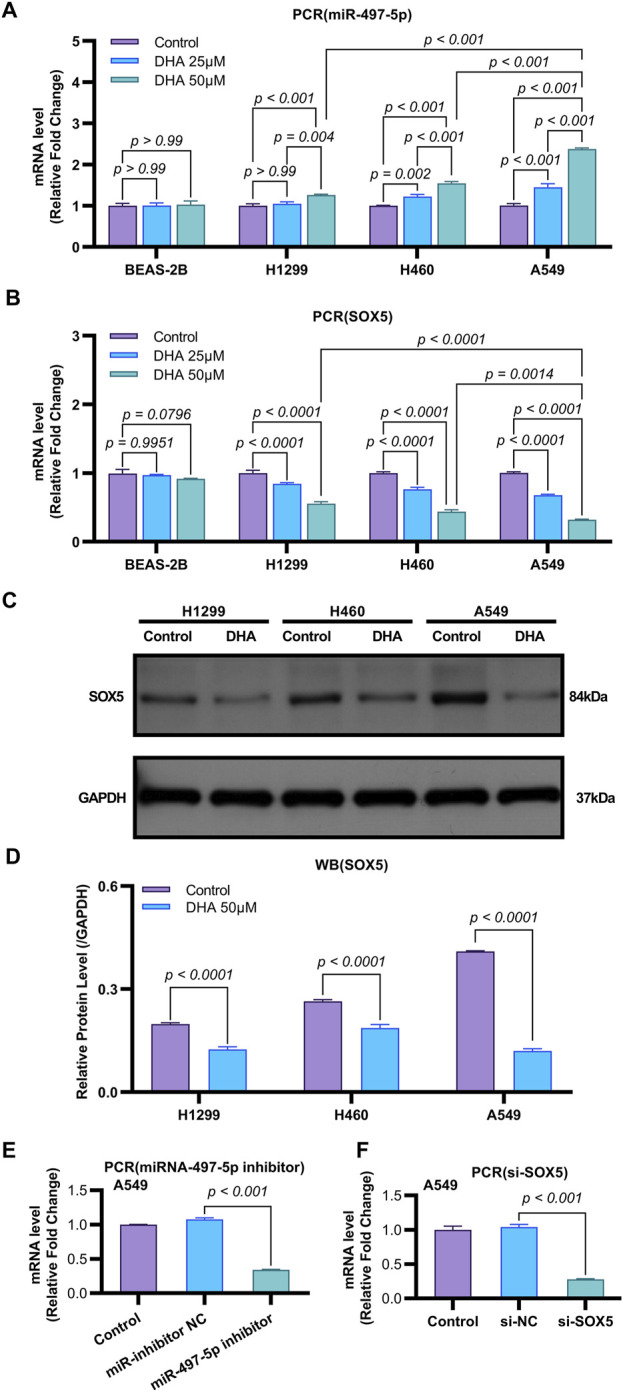
DHA Modulates the Expression of miR-497-5p and SOX5 in Non-Small Cell Lung Cancer. **(A)** Quantitative RT-PCR analysis of miR-497-5p levels in several lung cancer cell lines (H1299, H460, and A549) and normal human bronchial epithelial cells (BEAS-2B) after treatment with 25 and 50 μM DHA for 24 h. Data normalized to U6 snRNA. **(B)** Quantitative RT-PCR analysis of SOX5 levels in several lung cancer cell lines (H1299, H460, and A549) and normal human bronchial epithelial cells (BEAS-2B) after treatment with 25 μM and 50 μM DHA for 24 h. Data normalized to U6 snRNA. **(C,D)** Western blot analysis of SOX5 levels in several lung cancer cell lines (H1299, H460, and A549) after treatment with 25 μM and 50 μM DHA for 24 h. GAPDH served as endogenous control. **(E)** miR-497-5p inhibition efficiency: Transfection with miR-497-5p inhibitor (vs. NC inhibitor) significantly reduced basal miR-497-5p levels in A549 cells, validating functional knockdown. Data normalized to U6 snRNA. **(F)** SOX5 silencing verification: siRNA-mediated SOX5 knockdown (si-SOX5) significantly decreased SOX5 mRNA versus si-NC controls (*p* < 0.001), confirming target efficiency. Data normalized to U6 snRNA. All Quantitative RT-PCR data represent mean ± SD of three independent experiments. Western blots show representative images from biological triplicates.

Emerging evidence indicates that miR-497-5p plays a regulatory role in NSCLC cell proliferation, migration, invasion, and apoptosis (Huang et al., 2019; Tang et al., 2022). However, the relationship between DHA and miR-497-5p in NSCLC remains unexplored. To address this, we first analyzed miR-497-5p expression in five human lung adenocarcinoma (LAC) patient samples and a panel of cell lines. Quantitative RT-PCR analysis demonstrated significantly lower transcript levels of miR-497-5p in tumor tissues compared to paired non-tumorous counterparts (*p* = 0.006; Figure 3B). Consistently, we measured miR-497-5p expression in NSCLC cell lines (H1299, H460 and A549) and normal human bronchial epithelial cells (BEAS-2B) using qRT-PCR. miR-497-5p was significantly downregulated in H1299, H460 and A549 cells compared to BEAS-2B cells (*p* < 0.001, Figure 3G), consistent with previous studies. Subsequently, NSCLC cell lines (H1299, H460, A549) and the normal human bronchial epithelial cell line BEAS-2B were treated with 25 μM or 50 μM DHA for 24 h, followed by assessment of miR-497-5p expression via RT-PCR. Significant upregulation of miR-497-5p was observed in all three NSCLC cell lines following DHA treatment. This effect was most pronounced in A549 cells exposed to 50 μM DHA (Figure 4A), suggesting a potential tumor-suppressive role for miR-497-5p in NSCLC.

**FIGURE 5:**
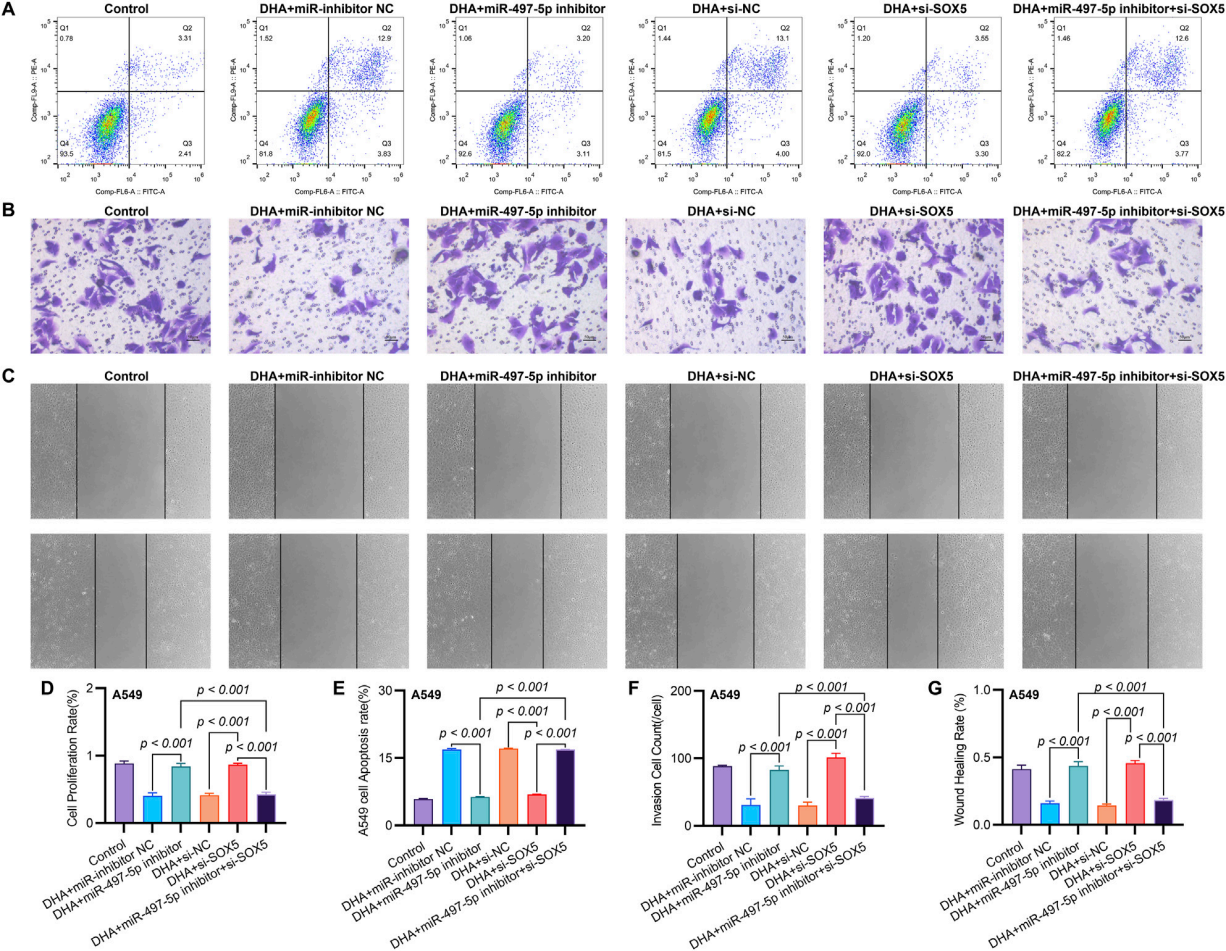
SOX5 Silencing Partially Reverses DHA-Mediated Suppression of Malignant Phenotypes via the miR-497-5p/SOX5 Axis. **(A)** Apoptosis analysis by Annexin V/PI flow cytometry: Representative dot plots showing apoptotic rates in A549 cells under indicated conditions. **(B)** Transwell invasion assay: Matrix-coated membranes visualize invasive capabilities (crystal violet staining). **(C)** Wound healing migration assay: Scratched monolayers monitored at 0/24 h demonstrates migratory capacity. **(D)** Cell viability quantification: CCK-8 absorbance measurements at 450 nm after 24 h treatments. **(E–G)** Quantitative analyses of apoptosis **(E)**, invasion **(F)**, and migration **(G)** data presented in **(A–C)**. Data expressed as mean ± SD (*n* = 3).

**FIGURE 6:**
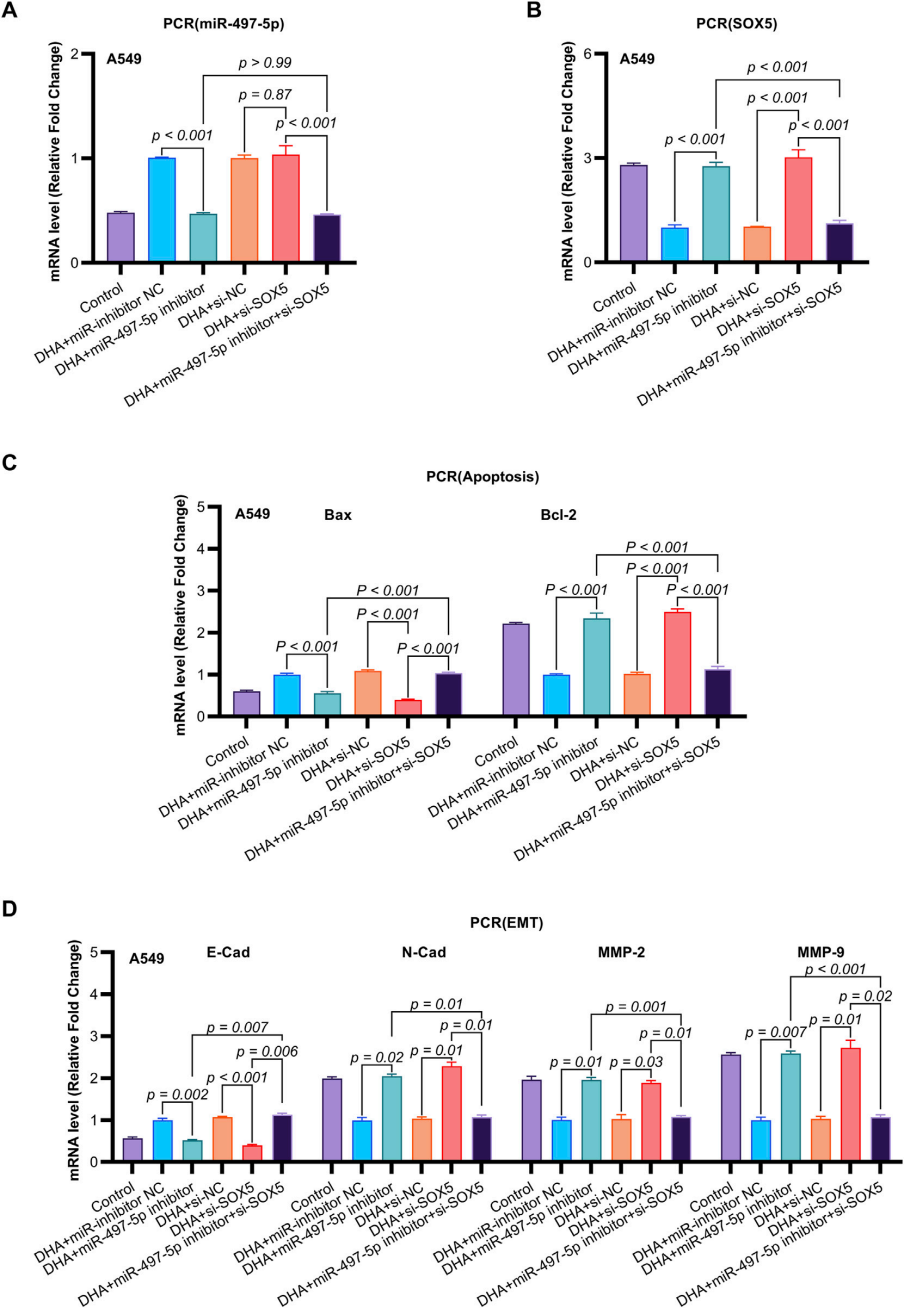
The Impact of DHA on the Expression of EMT and Apoptosis Mmarkers, Mediated Through the Regulation of the miR-497-5p/SOX5 Aaxis, was Assessed Using qRT-PCR. **(A)** miR-497-5p regulation hierarchy: qRT-PCR analysis confirmed DHA-induced miR-497-5p upregulation was effectively abolished by miR-497-5p inhibitor, while SOX5 knockdown (si-SOX5) showed no regulatory effect. Normalized to U6 snRNA. **(B)** Unidirectional targeting validation: miR-497-5p inhibition significantly elevated SOX5 mRNA expression, confirming SOX5 as downstream target. U6 served as endogenous control. **(C)** Apoptotic marker rescue: miR-497-5p inhibitor reversed DHA-mediated pro-apoptotic effects - suppressing Bax induction (*p* < 0.001) and restoring Bcl-2 expression. These alterations were rescued by concurrent SOX5 knockdown. **(D)** EMT marker reprogramming: Inhibition of miR-497-5p antagonized DHA’s anti-EMT effects, elevating mesenchymal markers N-Cadherin/MMP-2/MMP-9 while suppressing epithelial E-Cadherin. SOX5 co-silencing restored the DHA-induced anti-EMT phenotype. Data represent mean ± SD of triplicate experiments.

**FIGURE 7:**
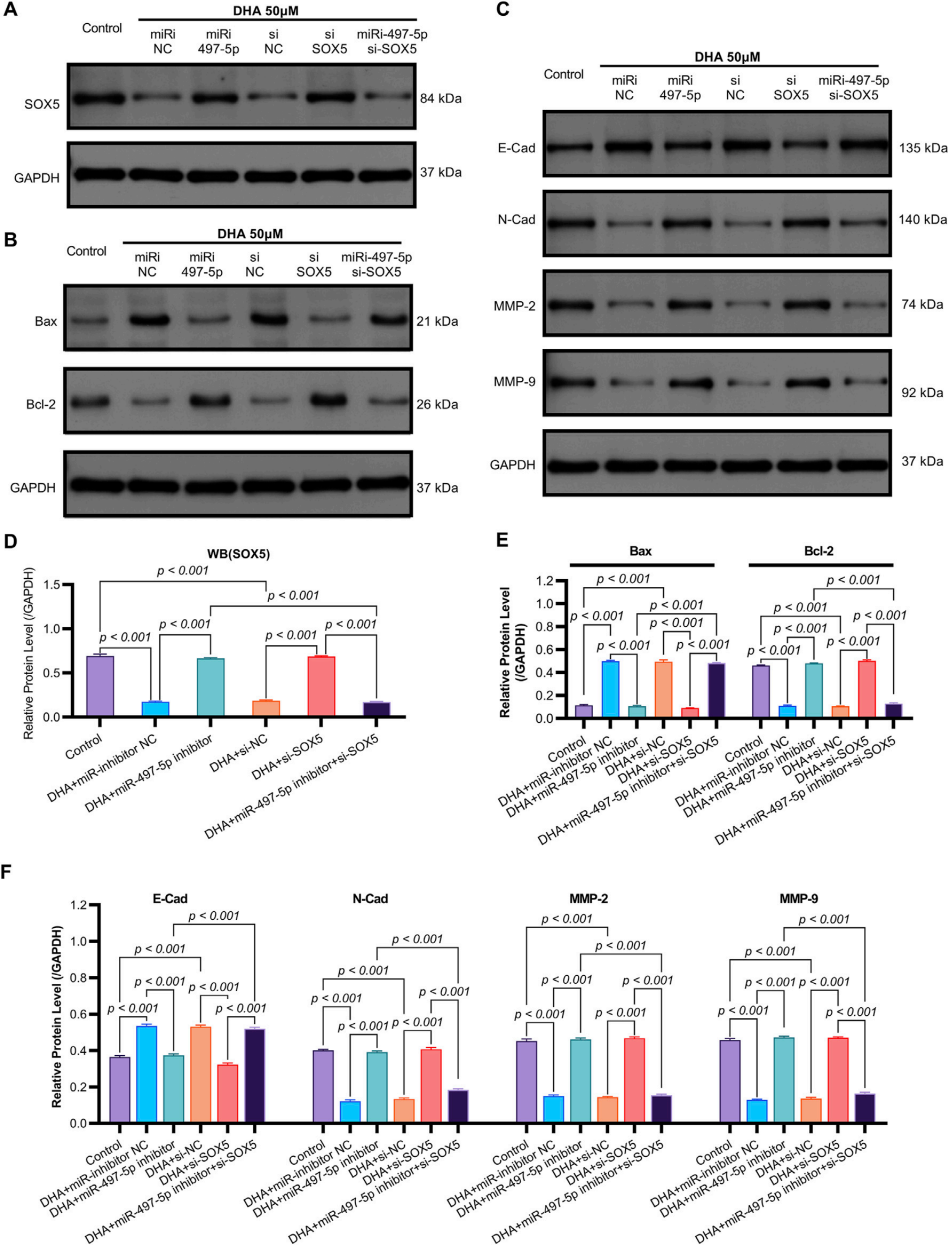
The Impact of DHA on the Expression of EMT and Apoptosis Markers, Mediated Through the Regulation of the miR-497-5p/SOX5 Axis, was Assessed Using Western blot. **(A)** SOX5 regulation cascade: Representative Western blots demonstrating DHA-induced SOX5 downregulation and its rescue by miR-497-5p inhibition. GAPDH loading control shown. **(B)** Densitometric quantification of SOX5 protein expression (normalized to GAPDH): Confirmed significant SOX5 reduction by DHA reversed by miR-497-5p inhibitor, while SOX5 knockdown validated targeting efficiency. **(C)** EMT marker reprogramming: Immunoblotting of epithelial (E-Cadherin) and mesenchymal (N-Cadherin, MMP-2, MMP-9) markers showing DHA-mediated phenotype reversal. **(D)** Quantitative analysis of EMT markers: miR-497-5p inhibition significantly antagonized DHA’s effects - reducing E-Cadherin while elevating N-Cadherin, MMP-2 and MMP-9. These alterations were rescued by SOX5 co-silencing. **(E)** Apoptotic regulator modulation: Protein expression of pro-apoptotic Bax and anti-apoptotic Bcl-2 under experimental conditions. **(F)** Densitometric analysis: miR-497-5p inhibitor reversed DHA-induced Bax upregulation and Bcl-2 downregulation. Concurrent SOX5 depletion restored pro-apoptotic signaling. Data represent mean ± SD of triplicate experiments.

To further investigate the functional role of miR-497-5p, A549 cells were transfected with a miR-497-5p inhibitor or negative control (miR-inhibitor NC) prior to 50 μM DHA treatment. qRT-PCR confirmed the transfection efficiency (*p* < 0.001, Figure 4E). The miR-497-5p inhibitor significantly reversed the DHA-induced upregulation of miR-497-5p (*p* < 0.001, Figure 6A) and attenuated the inhibitory effects of DHA on cell viability, migration, and invasion, as well as its pro-apoptotic effects (Figures 5A–G). Silencing miR-497-5p also reversed DHA-induced changes in EMT and apoptosis-related markers at both mRNA and protein levels (Figures 6C,D, 7B,C,E,F). These findings demonstrate that miR-497-5p is upregulated by DHA and mediates its anti-cancer effects in A549 cells.

### 3.3 SOX5 mediates the anti-cancer effects of dihydroartemisinin in NSCLC cells

To validate SOX5 levels in lung adenocarcinoma (LAC), we analyzed its expression in five paired LAC tissues and a panel of cell lines. Immunohistochemical (IHC) analysis revealed cytoplasmic SOX5 localization in all LAC and adjacent non-tumorous tissues, with significantly higher expression in all five LAC specimens versus paired non-tumorous counterparts (*p* < 0.001; Figures 3A–C). Consistent with protein findings, quantitative RT-PCR demonstrated elevated SOX5 transcripts in tumors relative to matched controls (*p* < 0.001; Figure 3E), while Western blotting confirmed SOX5 overexpression in tumor tissues compared to weak expression in non-tumorous tissues (*p* < 0.001; Figure 3F). This expression pattern was recapitulated *in vitro*, where NSCLC cell lines (H1299, H460, A549) exhibited significantly upregulated SOX5 mRNA and protein levels versus the normal bronchial epithelial cell line BEAS-2B (*p* < 0.001; Figures 3H,I). Treatment with 25 μM or 50 μM DHA for 24 h induced dose-dependent SOX5 downregulation in NSCLC lines, most pronounced in A549 cells at 50 μM (*p* < 0.001; Figure 4B), while SOX5 expression remained unaltered in BEAS-2B cells (*p* > 0.99; Figure 4B) - a finding corroborated by Western blotting showing significant SOX5 suppression post-50 μM DHA treatment in all NSCLC lines (*p* < 0.0001; Figures 4C,D). To mechanistically interrogate SOX5’s functional role, siRNA-mediated knockdown prior to 50 μM DHA treatment partially reversed DHA-mediated effects in A549 cells(Figure 4F), attenuating: (i) suppression of viability, migration, and invasion; (ii) induction of apoptosis (Figures 5A–G); and (iii) DHA-induced alterations in EMT and apoptosis-related markers at mRNA/protein levels (Figures 6B–D, 7A–F). Collectively, these results demonstrate that SOX5 – downregulated by DHA–mediates its anticancer effects in NSCLC cells.

### 3.4 Dihydroartemisinin regulates NSCLC cell behavior via the miR-497-5p/SOX5 axis

Previous studies have identified SOX5 as a target gene of miR-497-5p (He et al., 2020). qRT-PCR analysis showed that miR-497-5p knockdown significantly increased SOX5 expression in A549 cells (*p* < 0.001, Figures 6B, 7A,D), whereas SOX5 silencing did not alter miR-497-5p levels (*p* = 0.87, Figure 6A), confirming that miR-497-5p targets SOX5.

Co-transfection of A549 cells with miR-497-5p inhibitor and si-SOX5 restored the inhibitory effects of DHA on cell viability, migration, invasion, and apoptosis (Figures 5A–G). Furthermore, qRT-PCR and Western blot analyses demonstrated that miR-497-5p inhibition elevated N-Cadherin, MMP-2, MMP-9, and Bcl-2 levels while reducing E-Cadherin and Bax expression, which were reversed by SOX5 depletion (Figures 6A–D, 7B–F). These findings suggest that DHA inhibits NSCLC progression by modulating the miR-497-5p/SOX5 axis.

### 3.5 Dihydroartemisinin suppresses tumor growth *in vivo* via the miR-497-5p/SOX5 axis

**FIGURE 8:**
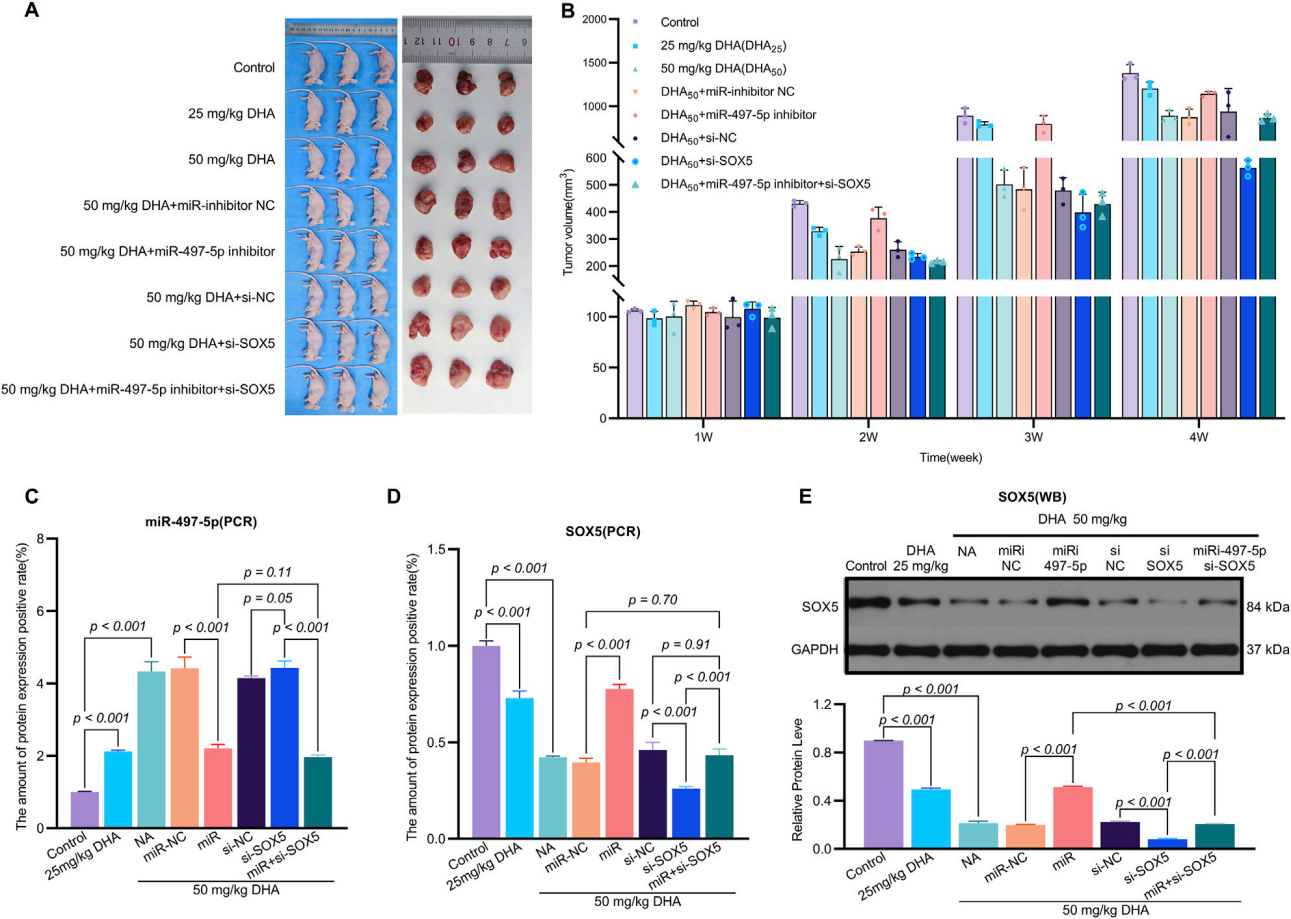
Dihydroartemisinin Suppresses Tumor Growth *In Vivo* via the miR-497-5p/SOX5 Axis. **(A)**. Experimental design of xenograft tumor model. A549 cells transfected with miR-inhibitor NC (negative control), miR-497-5p inhibitor, si-NC (scrambled siRNA), si-SOX5, or miR-497-5p inhibitor + si-SOX5 were subcutaneously injected into nude mice. Mice were treated with 25 or 50 mg/kg DHA (intraperitoneal injection, every 2 days) for 4 weeks. **(B)** DHA dose-dependently reduces tumor weight. Final tumor weights (measured post-sacrifice) show significant suppression by both 25 mg/kg and 50 mg/kg DHA. This effect was reversed by miR-497-5p inhibition or SOX5 knockdown, while co-treatment with miR-497-5p inhibitor + si-SOX5 restored DHA’s antitumor efficacy. **(C)** Dynamic tumor volume changes during treatment. Tumor volume was measured every 7 days. 50 mg/kg DHA showed the strongest growth inhibition. Key reversal groups: miR-497-5p inhibitor: Abrogated DHA-induced suppression. si-SOX5: Partially reversed DHA efficacy. miR-497-5p inhibitor + si-SOX5: Synergistically restored tumor suppression. **(D)** SOX5 mRNA expression in tumor tissues. qRT-PCR analysis confirmed DHA significantly downregulated SOX5, consistent with *in vitro* data. SOX5 knockdown (si-SOX5) further reduced its expression, validating targeting efficiency. **(E)** miR-497-5p expression in tumor tissues. qRT-PCR revealed DHA (50 mg/kg) markedly upregulated miR-497-5p. Transfection with miR-497-5p inhibitor effectively blocked this induction, confirming functional antagonism. Data represent mean ± SD of triplicate experiments.

To evaluate the *in vivo* efficacy of DHA, we established a xenograft mouse model where treatment with 25 mg/kg and 50 mg/kg DHA significantly suppressed tumor growth (Figures 8A,B). These doses were rigorously selected based on clinical translatability considerations. Preclinically, our regimen aligns with established NSCLC xenograft studies, including doses of 60 mg/kg (5×/week) (Tong et al., 2016) and 50–100 mg/kg/day (Dai et al., 2014) in A549 models, confirming robust antitumor activity within this range. Clinically, DHA exhibits exceptional tolerability in humans, evidenced by malaria trials (>15,000 patients) reporting no significant toxicity at therapeutic doses (Ribeiro and Olliaro, 1998; Adjuik et al., 2004), and an ongoing NSCLC trial (NCT03402464) safely administering escalating doses up to 80 mg twice daily. Dose conversion feasibility is demonstrated as the Human Equivalent Dose (HED) for mouse 50 mg/kg is −4 mg/kg (body surface area scaling), far below the clinically tested 80 mg/day (≈1.14 mg/kg for a 70 kg adult); plasma concentrations from human studies achieve >30 μM, matching our *in vitro* 50 μM efficacy threshold. Furthermore, emerging nanoscale carriers (e.g., PEGylated liposomes, Fe^2+^-MOFs) (Liu et al., 2015; Jia et al., 2021) enhance tumor targeting and prolong circulation, potentially reducing effective doses by 5–10-fold. For instance, DHA-loaded nano-liposomes increased tumor accumulation >8-fold while halving required doses (Li H. et al., 2019; Shen et al., 2020).

**FIGURE 9:**
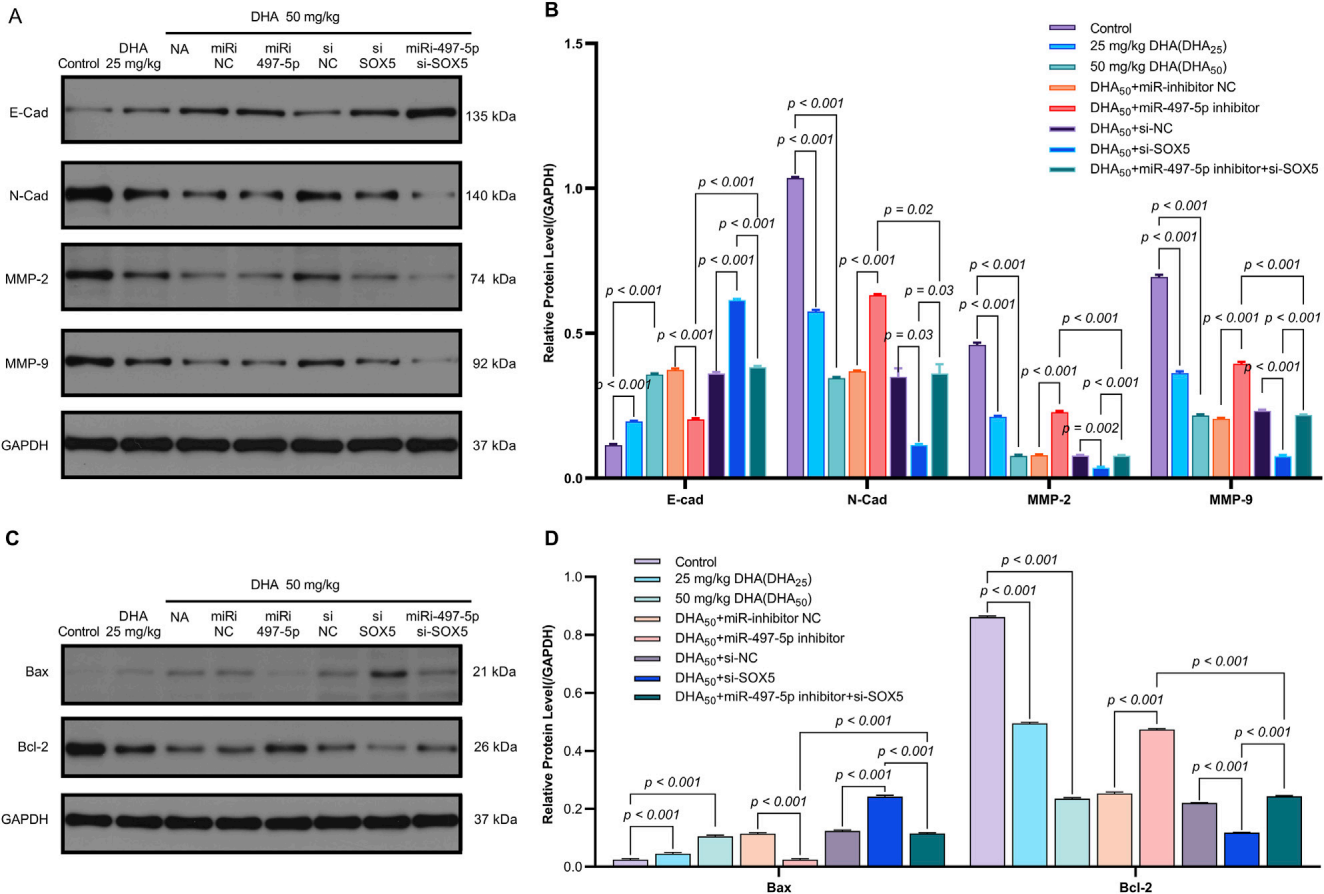
Dihydroartemisinin Modulates the Expression of EMT and Apoptosis Markers *In Vivo* via the miR-497-5p/SOX5 Axis. **(A,B)** DHA reverses EMT progression in tumor tissues. Western blot analysis of epithelial-mesenchymal transition (EMT) markers. Epithelial marker: E-Cadherin (E-Cad) was upregulated by DHA.Mesenchymal markers: N-Cadherin (N-Cad), MMP-2, and MMP-9 were downregulated by DHA.Reversal effects: miR-497-5p inhibition or SOX5 knockdown attenuated DHA’s modulation of EMT markers, while dual inhibition (miR-497-5p inhibitor + si-SOX5) restored DHA’s efficacy. **(C,D)** DHA promotes pro-apoptotic signaling. Western blot of apoptosis regulators: Pro-apoptotic Bax was increased by 50 mg/kg DHA. Anti-apoptotic Bcl-2 was suppressed by DHA. Bax/Bcl-2 ratio significantly rose in DHA groups, Bax/Bcl-2 ratio (apoptosis indicator) significantly rose in DHA groups. Key trend: miR-497-5p/SOX5 axis disruption blunted DHA-induced apoptosis, rescued by co-targeting both molecules. Data represent mean ± SD of triplicate experiments.

The miR-497-5p inhibitor and si-SOX5 reversed these effects, whereas co-treatment with miR-497-5p inhibitor and si-SOX5 restored tumor suppression (Figures 8A,B). DHA treatment increased miR-497-5p expression and decreased SOX5 levels in tumor tissues, consistent with *in vitro* findings (Figures 8C–E). Additionally, changes in EMT and apoptosis-related markers were observed in the tumor model (Figures 9A–D). These results demonstrate that DHA inhibits tumor growth *in vivo* by regulating the miR-497-5p/SOX5 axis.

## 4 Discussion

The results of this study are consistent with and extend previous findings on the anticancer effects of dihydroartemisinin (DHA) in non-small cell lung cancer (NSCLC). Similar to prior studies, our findings demonstrate that DHA significantly inhibits cell viability, migration, and invasion while promoting apoptosis in NSCLC cells (Rani et al., 2025; Jiang et al., 2016; Chen et al., 2017). For example, Liao et al. (2020) reported that DHA induced apoptosis in A549 cells via the mitochondrial pathway, characterized by the upregulation of Bax and downregulation of Bcl-2, which aligns with our observations (Rani et al., 2025). However, our study uniquely identifies the miR-497-5p/SOX5 axis as a key regulatory mechanism underlying DHA’s anticancer effects, a pathway not previously explored in the context of NSCLC. This novel insight distinguishes our study from existing literature and deepens the understanding of DHA’s molecular mechanisms.

The upregulation of miR-497-5p by DHA and its subsequent inhibition of SOX5 expression represent a significant advancement in elucidating the anticancer properties of DHA. Our results confirm that inhibition of miR-497-5p reverses DHA’s effects on cell viability, migration, and apoptosis. Previous studies have shown that miR-497-5p functions as a tumor suppressor in various cancers (Li et al., 2017), a role consistent with its function in NSCLC as revealed in this study. Additionally, our identification of SOX5 as a downstream target of miR-497-5p provides a mechanistic link between DHA and its anticancer effects. SOX5, a transcription factor involved in epithelial-mesenchymal transition (EMT) and metastasis, has been shown to promote tumor progression in other cancers (Chen X. et al., 2018; Hu et al., 2018). Our findings suggest that DHA disrupts this pro-cancer pathway, potentially offering a therapeutic strategy for NSCLC.

The effective *in vitro* concentration range of dihydroartemisinin (DHA) against lung cancer is well-documented across multiple studies, spanning 5–160 μM (Li G. et al., 2019). Our selection of 50 μM aligns with concentrations employed in investigations by Mi YJ, Zhou et al., and others (Li G. et al., 2019; Wen et al., 2024; Wang et al., 2024). While lower concentrations (<50 μM) are utilized in certain studies, Lin et al. specifically determined the IC_50_ of free DHA in A549 cells to be 25–40 μM (CCK-8 assay), with 50 μM inducing apoptosis in >80% of cells (Dai et al., 2014). Our data corroborate this concentration-dependent efficacy: treatment with 25 μM DHA for 24 h yielded a 1.5-fold increase in miR-497-5p expression and a 32% reduction in SOX5 protein levels in A549 cells. In contrast, 50 μM DHA elicited a substantially enhanced response, elevating miR-497-5p expression by 2.4-fold and diminishing SOX5 protein by 68%. This marked differential effect identifies 50 μM as the optimal threshold for robust activation of the miR-497-5p/SOX5 axis under these experimental conditions. We fully acknowledge the pharmacokinetic constraints of free DHA in humans, as evidenced by Ericsson et al. reporting a peak plasma concentration (C_max_) of only 9.5 ± 4.1 μM following a single 200 mg oral dose (t_1/2_ > ≈ 1.1 h, near-complete clearance within 24 h), and Morris et al. demonstrating sustained plasma levels <15 μM even after repeated dosing-concentrations insufficient to approach the 50 μM threshold (Ericsson et al., 2014; Morris et al., 2011). Consequently, the 50 μM concentration employed in this study is strictly for *in vitro* mechanistic investigation, serving to establish the effective concentration required for DHA-mediated modulation of the miR-497-5p/SOX5 pathway, rather than to simulate physiological exposure.

Dihydroartemisinin (DHA), a reduced derivative of artemisinin, contains a critical peroxide bridge within its core structure (Wang et al., 2023). This bridge undergoes Fe^2+^-dependent cleavage in the high ferrous iron environment characteristic of tumor cells, generating substantial reactive oxygen species (ROS) and triggering an oxidative stress cascade (Cao et al., 2016). Compared to artemisinin, DHA exhibits enhanced water solubility and cellular permeability due to C-10 hydroxyl substitution, facilitating its bioactivation within cancer cells. Consistent with this, DHA has been extensively documented to induce significant oxidative stress in diverse cancer cell types, including non-small cell lung cancer (NSCLC) (Zhang et al., 2021). Furthermore, oxidative stress is a well-established key regulator of microRNA (miRNA) expression (Pelizzaro et al., 2024), as elevated intracellular ROS levels can modulate specific signaling pathways, thereby influencing the transcription or maturation of various miRNAs (Kura et al., 2024; Thulasingam et al., 2011; Peng et al., 2023). Therefore, if ROS serve as the primary or essential signaling molecules mediating the observed effects of DHA on miRNA expression, the application of potent ROS scavengers (e.g., N-acetylcysteine (NAC), Tempol, or catalase overexpression) should significantly attenuate or abrogate the DHA-induced upregulation of miR-497-5p. However, direct experimental validation using such ROS scavengers to antagonize DHA’s effect has not yet been performed in the current stage of this investigation. Validating this mechanistic link constitutes a critical step for precisely defining DHA’s mode of action and represents a current limitation of this study. In prostate cancer models, DHA significantly downregulates expression of UHRF1 (Ubiquitin-like protein) and DNMT1. UHRF1 serves as a critical cofactor for DNMT1, orchestrating its recruitment to hemimethylated DNA sites (Si-hao et al., 2022). DHA-mediated attenuation of UHRF1/DNMT1 complex activity promotes promoter demethylation of specific genes (Si-hao et al., 2022). As miR-497-5p functions as a tumor-suppressive miRNA, its promoter may undergo analogous demethylation, alleviating transcriptional repression (Chen et al., 2024). Intriguingly, DHA exhibits context-dependent epigenetic effects: in systemic lupus erythematosus (SLE) models, it upregulates DNMT1 and enhances global DNA methylation (Chen et al., 2018). This dichotomy suggests tissue/disease-specific modulation, potentially mediated through indirect signaling pathways (e.g., NF-κB inhibition) influencing DNMT activity (Li et al., 2014). Hypermethylation of the miR-497-5p promoter correlates inversely with its expression in gastric and hepatocellular carcinomas, as evidenced by significantly lower miR-497-5p levels in hypermethylated hepatocellular carcinoma cell lines (e.g., SNU423/182) versus hypomethylated counterparts (Chen et al., 2024; Tian et al., 2021). While direct experimental validation of DHA’s impact on miR-497-5p promoter methylation or histone modifications remains unreported, existing evidence supports a plausible mechanistic framework: DHA may induce miR-497-5p promoter demethylation via suppression of the UHRF1/DNMT1 complex, concurrently modulating histone-modifying enzyme activity through pathways such as NF-κB/MAPK to reverse epigenetic silencing. Future investigations should directly assess DHA’s effects on chromatin dynamics at the miR-497-5p locus in disease-relevant models and explore its synergistic interactions with tumor-suppressive pathways (Peng et al., 2021).

This study underscores the potential of DHA as a multi-target therapeutic agent for NSCLC. By regulating the miR-497-5p/SOX5 axis, DHA not only inhibits tumor growth but also suppresses metastasis and induces apoptosis. These findings are particularly significant given the limited efficacy of current therapeutic agents for advanced NSCLC. For example, DHA’s ability to reverse EMT markers (e.g., upregulation of E-Cadherin and downregulation of N-Cadherin, MMP-2, and MMP-9) suggests its potential to prevent tumor spread, a major challenge in NSCLC treatment (Kalia, 2015; Davis et al., 2014). Future studies could explore combining DHA with existing therapies, such as immune checkpoint inhibitors, to enhance treatment efficacy.

Our results provide a solid foundation for further research into the clinical application of DHA. The *in vivo* inhibitory effects of DHA on tumors through the miR-497-5p/SOX5 axis highlight its potential as a targeted therapy. For instance, reductions in tumor volume and weight observed in xenograft models treated with DHA demonstrate its efficacy in a physiological setting (Jiang et al., 2016; Tong et al., 2016). These findings could pave the way for clinical trials evaluating DHA as monotherapy or adjuvant therapy for NSCLC patients, especially those resistant to conventional treatments.

While this study provides valuable insights, several limitations should be acknowledged. Firstly, the research primarily focused on A549 cells. Although this cell line is a valuable model, it may not fully represent the heterogeneity of NSCLC, and the findings lacked validation with clinical samples. While the present study focused primarily on the A549 cell line as a model of non-small cell lung cancer (NSCLC), this selection was strategically employed to achieve mechanistic depth in investigating the ROS/miR-497-5p/SOX5 axis. A549 represents a well-validated adenocarcinoma model harboring characteristic KRAS mutations and, critically, exhibits dysregulated iron metabolism-a pathophysiological feature essential for DHA bioactivation that mirrors clinical NSCLC profiles (Zheng et al., 2023; Ba et al., 2012). Our data robustly demonstrate that DHA-induced oxidative stress orchestrates miR-497-5p-mediated SOX5 suppression in this system. Although validation across diverse cellular contexts would further strengthen generalizability, three lines of evidence support the biological relevance of our findings: 1) The prerequisite high intracellular Fe^2+^ levels for DHA activation are conserved across NSCLC subtypes (Zheng et al., 2023; Ishikado et al., 2013); 2) miR-497-5p functions as an evolutionarily conserved stress-responsive regulator in epithelial malignancies (Chai et al., 2018); and 3) Core DHA responses (e.g., ROS-mediated cytotoxicity) exhibit significant inter-cell-line concordance in independent NSCLC datasets (Ni et al., 2024; Lai et al., 2023). Future investigations should prioritize examining this regulatory axis in physiologically relevant models incorporating tumor microenvironmental influences, such as patient-derived organoids or *in vivo* systems, where stromal interactions may modulate DHA sensitivity. Consequently, future studies should include a broader range of cell lines and patient-derived xenografts to validate these findings. Additionally, clinical sample studies are needed to validate the expression levels of miR-497-5p and SOX5 in NSCLC patients and their correlation with prognosis, while clinical trials should be initiated to evaluate the safety and efficacy of DHA in NSCLC treatment. Secondly, the exact mechanism by which DHA upregulates miR-497-5p remains unclear. Investigating the upstream regulators of miR-497-5p could provide a more comprehensive understanding of DHA’s role in this regulatory pathway. Finally, the *in vivo* experiments were conducted in immunocompromised mice. While providing essential proof-of-concept, this model may not fully reproduce the complex tumor microenvironment, including immune interactions, found in human patients. Incorporating immunocompetent models could therefore offer more physiologically relevant insights into the effects of DHA on tumor-immune interactions.

## 5 Conclusion

In conclusion, this study elucidates the role of the miR-497-5p/SOX5 axis in mediating the anticancer effects of DHA on NSCLC *in vitro*. Our findings offer novel insights into the anticancer potential of DHA in NSCLC by uncovering this key molecular mechanism. While these results establish the fundamental molecular basis for DHA’s action, it is acknowledged that the concentrations used *in vitro* are currently beyond those readily achievable clinically. Therefore, this work primarily provides significant mechanistic groundwork and highlights a potential therapeutic axis (miR-497-5p/SOX5) for future exploration. Future studies must rigorously address the identified limitations, particularly bridging the gap between *in vitro* efficacy and clinically feasible dosing strategies, to validate and optimize DHA’s potential application.

## Data Availability

The original contributions presented in the study are included in the article/supplementary material, further inquiries can be directed to the corresponding author.

## Abbreviations

DHA, dihydroartemisinin; EMT, Epithelial-mesenchymal transition; LC, Lung cancer; LAC, lung adenocarcinoma; NSCLC, Non-small cell lung cancer.
